# Whole-Genome Analyses Reveal Genomic Characteristics and Selection Signatures of Lincang Humped Cattle at the China–Myanmar Border

**DOI:** 10.3389/fgene.2022.833503

**Published:** 2022-03-22

**Authors:** Luyang Sun, Kaixing Qu, Xiaohui Ma, Quratulain Hanif, Jicai Zhang, Jianyong Liu, Ningbo Chen, Quji Suolang, Chuzhao Lei, Bizhi Huang

**Affiliations:** ^1^ Yunnan Academy of Grassland and Animal Science, Kunming, China; ^2^ Key Laboratory of Animal Genetics, Breeding and Reproduction of Shaanxi Province, College of Animal Science and Technology, Northwest A&F University, Yangling, China; ^3^ Academy of Science and Technology, Chuxiong Normal University, Chuxiong, China; ^4^ National Institute for Biotechnology and Genetic Engineering, Faisalabad, Pakistan; ^5^ Institute of Animal Science, Tibet Academy of Agricultural and Animal Husbandry Science, Lhasa, China

**Keywords:** whole-genome resequencing, Lincang humped cattle, genetic characteristics, selection signatures, *HELB*

## Abstract

The location on the Yunnan border with Myanmar and its unique cultural landscape has shaped Lincang humped cattle over time. In the current study, we investigated the genetic characteristics of 22 Lincang humped cattle using whole-genome resequencing data. We found that Lincang humped cattle derived from both Indian indicine and Chinese indicine cattle depicted higher levels of genomic diversity. Based on genome-wide scans, candidate genomic regions were identified that were potentially involved in local thermal and humid environmental adaptions, including genes associated with the body size (*TCF12*, *SENP2*, *KIF1C*, and *PFN1*), immunity (*LIPH*, *IRAK3*, *GZMM*, and *ELANE*), and heat tolerance (*MED16*, *DNAJC8*, *HSPA4*, *FILIP1L*, *HELB*, *BCL2L1*, and *TPX2*). Missense mutations were detected in candidate genes *IRAK3*, *HSPA4*, and *HELB*. Interestingly, eight missense mutations observed in the *HELB* gene were specific to the indicine cattle pedigree. These mutations may reveal differences between indicine and taurine cattle adapted to variable climatic conditions. Our research provides new insights into the genetic characteristics of Lincang humped cattle representing Lincang and Pu’er areas as an important channel for the migration of Indian indicine from domestication centers toward southwestern China.

## Introduction

Domestic cattle comprise two subspecies, humpless taurine (*Bos taurus*) and humped indicine or zebu (*Bos indicus*), both of which are derived from extinct wild aurochs (*Bos primigenous*) (Decker et al., 2014). Existing research has recognized that worldwide cattle can be divided into five continental groups, European taurine, Eurasian taurine, East Asian taurine, Chinese indicine, and Indian indicine, through whole-genome sequencing analysis (Chen et al., 2018). As for current distribution patterns, modern cattle live in different geographical and climatic zones worldwide. Taurine cattle mainly inhabit temperate environments. In contrast, indicine cattle adapt to continuous high and variable temperate climates (Barendse, 2017).

Yunnan province in China is traversed by the Tropic of Cancer, which is mainly a tropical and subtropical climate zone. Recent studies have identified the complex genetic diversity and admixture patterns of cattle breeds in Yunnan province (Chen et al., 2018; R.; Li et al., 2019; Liu et al., 2020). Lincang humped cattle is the more primitive regional livestock breed mainly distributed in the southern part of Lincang and Pu’er cities in Yunnan province bordering Myanmar (Gan, 2011). The exact history of the formation of Lincang humped cattle has not been verified. However, the reason for the formation of this breed is the adaptive selection and breeding of local farmers according to their social needs. Moreover, the local ethnicity (Wa ethnic) regards cattle as a totem, and the birth of the Wa ethnicity culture is closely associated with cattle. Lincang humped cattle displays superior characteristics of heat tolerance and resistance to disease and are one kind of Yunnan high-humped cattle (Y. Zhang, 2011). Long-term strong natural selection and human-mediated selection might have potentially affected the structure of the Lincang humped cattle genome by forming detectable selection signals in functional genes (Andersson and Georges, 2004; Hoffmann, 2010).

The unique adaptive characterization of indigenous African and Asian cattle breeds has become a hot topic based on whole-genome sequencing. (Ben-Jemaa, Mastrangelo, Lee, Lee, and Boussaha, 2020; J.; Kim et al., 2017; Xia et al., 2021). In the current study, the whole genome of 22 Lincang humped cattle was resequenced and compared with the sequence data of 61 cattle from five continental groups. Our analysis reports the genome characterization of Lincang humped cattle using whole-genome resequencing, providing many insights into their candidate signatures of positive selection.

## Methods

### Samples and Resequencing

A total of 22 domestic Lincang humped cattle (NCBI: PRJNA781760) from Cangyuan Wa Ethnicity Autonomous County, Lincang City, Yunnan Province, China, were sequenced. The purity of that breed was ensured throughout the sampling. However, one cattle might fall under hybrid cattle. Genomic DNA was extracted from the ear tissue samples. Twenty-two paired-end DNA libraries were constructed for the 22 pieces (500 bp insert size) and subjected to Illumina NovaSeq sequencing at the Novogene Bioinformatics Institute, Beijing, China. The genome sequence data of 61 cattle from five continental groups including European cattle breeds [Angus and Simmental (*n* = 17)], Chinese native breeds (Leiqiong, Guangfeng, Jian, Jingjiang, Wannan, and Wenshan *n* = 24), Indian cattle breeds (Sahiwal, Hariana, Tharparkar, Nelore, Gir, and Brahman, *n* = 10), and Korean native breed (Hanwoo, *n* = 10) were used for the combined analysis (Supplementary Table S1).

### Reads Mapping and SNP Calling

The clean reads were mapped to the latest *Bos taurus* reference genome (ARS-UCD1.2_Btau5.0.1Y.fa) using BWA-MEM (0.7.13-r1126) (H. Li & Durbin, 2009). The average mapping rate of the reads was 99.72%, and the sequencing coverage was approximately 9.75 × (ranging from 8.94 to 11.78) per individual. Duplicate reads were removed using Picard Tools (http://broadinstitute.github.io/picard). The genome analysis toolkit (GATK, version 3.8) (Using the HaplotypeCaller, GenotypeGVCFs, and Select Variants module) was used to detect SNPs. Previous studies have referred to the SNP calling parameters (Chen et al., 2018). Moreover, ANNOVAR (Using the table_annovar.pl module) (K. Wang, Li, & Hakonarson, 2010) was used to annotate the functions of the SNPs.

### Population Genetic Analysis

VCFtools (Danecek et al., 2011) was used to estimate the nucleotide diversity of each breed in window sizes of 50 kb with 20 kb increment. Furthermore, the linkage disequilibrium (LD) decay between pairwise SNPs was calculated by PopLDdecay software (C. Zhang, Dong, Xu, He, & Yang, 2019). PLINK was used to detect the runs of homozygosity (ROH) in each cattle population. The number and length of ROH for each population were estimated and classified into three categories: 0.5–1 Mb, 1–2 Mb, and 2–4 Mb (Sun et al., 2021). PLINK (version 1.9) (Purcell et al., 2007) was again used to remove the linkage sites in genomic data (--indep-pair-wise 50 5 0.2) to perform principal component analysis (PCA) and ADMIXTURE analysis. To accurately identify the components of Lincang humped cattle, ADMIXTURE software (Alexander & Lange, 2011) was used to analyze the population structure with a kinship (K) set from 2 to 5. The aforementioned results were visualized *via* RStudio software (Loraine et al., 2015). A phylogenetic tree was constructed using the neighbor-joining (NJ) method by PLINK with the matrix of pairwise genetic distances and visualized in MEGA7 (Kumar, Stecher, & Tamura, 2016) and FigTree v1.4.3 (http://tree.bio.ed.ac.uk/software/figtree/).

### Selective Sweep Identification

To identify selective sweep regions in Lincang humped cattle, three methods were used (I) The fixation index (*F*_ST_) values (Weir & Cockerham, 1984; Porto-Neto et al., 2013), (II) Cross-population extended haplotype homozygosity (XP-EHH) (Sabeti et al., 2007), and (III) The composite likelihood ratio (CLR) (Nielsen et al., 2005). *F*_ST_ and XP-EHH were calculated with a 50-kb sliding window and 20-kb steps along the autosomes using VCFtools and in-house scripts between Lincang humped cattle and the reference group. The CLR test was calculated for sites in non-overlapping 50-kb windows using “SweepFinder”. Tajima’s D statistics and nucleotide diversity were calculated for each candidate gene using VCFtools. Furthermore, functional annotation (GO analysis) and KEGG pathway enrichment were performed by DAVID 6.8 (Huang da, Sherman, & Lempicki, 2009), and FDR <0.05 was used as a threshold to detect significantly enriched genes and pathways. The LD heatmap was visualized for candidate genes using VCFtools based on LDBlockShow (Dong et al., 2021).

## Results

### Resequencing, Identification, and Diversity of Single Nucleotide Polymorphisms

The Lincang humped cattle (*n* = 22) were selected for genome resequencing (Supplementary Figure S1A). The data were combined with the available dataset (*n* = 61) from 15 breeds, giving a total of 83 individuals (Supplementary Table S1). Nelore, Gir, and Brahman were also used to represent Indian indicine cattle in this study. In total, 2.58 billion clean reads were generated and aligned to the reference genome ARS-UCD1.2_Btau5.0.1Y.fa with an average alignment rate of 99.72% and an average depth of 11.87 × in Lincang humped cattle.

**FIGURE 1:**
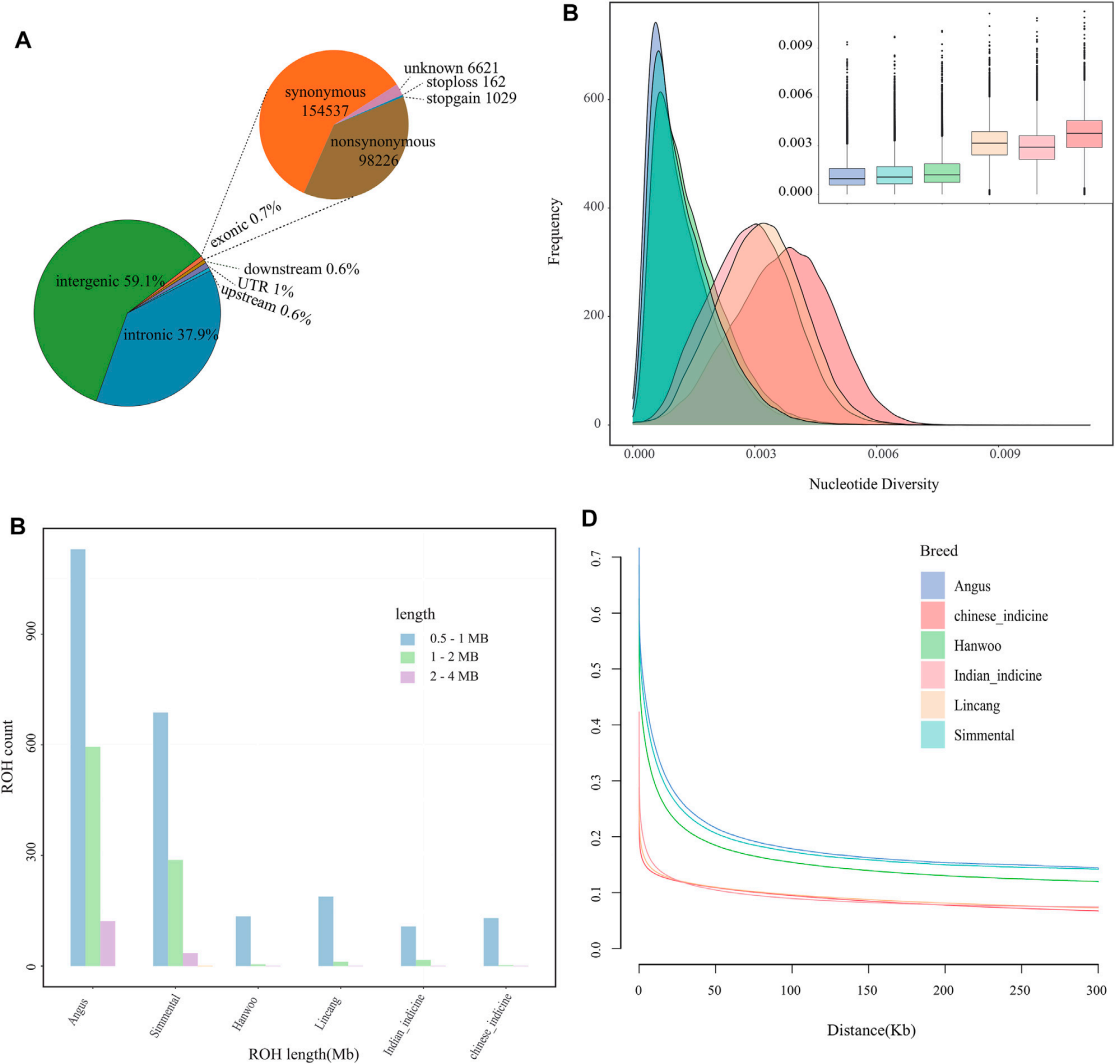
Genomic characteristics of cattle population. **(A)** Functional classification of the detected SNPs in Lincang cattle. **(B)** The estimation of number and length of ROH for each group. **(C)** Estimated nucleotide diversity for each group. **(D)** Linkage disequilibrium (r2) decay on cattle autosomes estimated from each group.

Furthermore, 34,636,187 SNPs were detected in mapped reads across 22 Lincang humped cattle. Approximately, 0.7% of SNPs with 98,226 nonsynonymous and 154,537 synonymous SNPs were detected in exonic regions. Then, 37.9% of SNPs were found in intronic regions, 59.1% were found in intergenic regions, 1% of SNPs were observed in untranslated regions (UTR), and 1.2% of SNPs were found in upstream and downstream of genes (Figure 1A). The highest number of SNPs was observed in the Chinese indicine, while the number was lower in taurine cattle than in indicine cattle (Supplementary Table S2). Moreover, the highest and second highest numbers of specific SNPs were found in Chinese indicine and Lincang humped cattle, respectively (Supplementary Figure S1B). The difference in the number of SNPs and specific SNPs might indicate the differences in the cattle numbers and different populations.

In the absence of pedigree records, ROH may help to infer the level of inbreeding. At the ROH threshold of >2 Mb, Lincang humped cattle showed low levels of genomic inbreeding. Cattle of European origin appeared to be more inbred than other groups (Figure 1B). The nucleotide diversity was the highest in Chinese indicine cattle, followed by Lincang and Indian indicine cattle (Figure 1C). The lowest nucleotide diversity was found in taurine cattle. The genome-wide LD was lower for the indicine cattle than for the taurine cattle, which might indicate faster LD decay in indicine cattle than in taurine cattle (Figure 1D).

### Phylogenetic Relationship, Principal Component Analysis, and Population Structure

**FIGURE 2:**
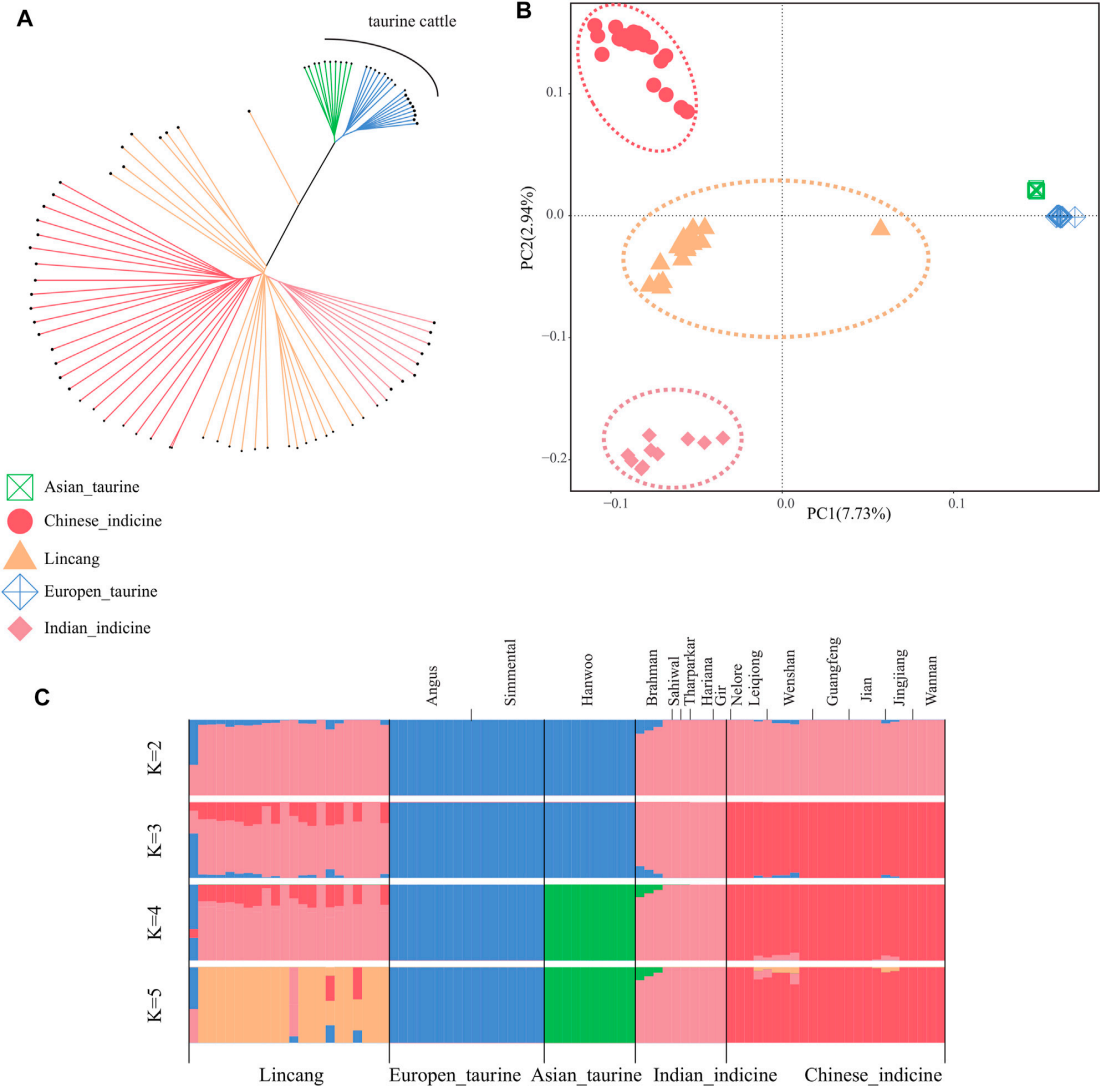
Population structure and relationships of Lincang cattle. **(A)** Neighbor-joining tree of the relationships between the cattle group. **(B)** Principal component analysis of the cattle group. **(C)** Model-based clustering of the cattle group using ADMIXTURE (with K = 2 to K = 5). Breeds are colored by geographic regions and labeled with breed names.

The phylogenetic relationship among 83 cattle samples was explored based on the autosomal SNPs. The NJ tree separated taurine and indicine in its clade. The taurine clade clustered Angus, Simmental, and Hanwoo, whereas Chinese indicine, Indian indicine, and Lincang humped cattle were clustered into the indicine clade (Figure 2A). An individual of Lincang humped cattle appeared alone between the taurine and the indicine clade, indicating it as a hybrid. Principal component analysis (PCA) demonstrated a clear genetic structure. PC1 explained 7.73% of the total variation and was driven by the difference between taurine and indicine cattle. Within indicine, a separation was found between Chinese indicine and Indian indicine along PC2. The Lincang humped cattle were found at an intermediate position between Chinese indicine and Indian indicine (Figure 2B). The admixture estimated from K = 2 to K = 5 showed gradual separation of Lincang humped cattle. When K = 2, the CV error value was the lowest, which means the most reasonable biological explanation was obtained. Lincang humped cattle belonged to *Bos indicus*, composed of crosses with Indian–Chinese indicine genotypes (Figure 2C). In particular, hybrid cattle between taurine and indicine cattle appeared in Lincang humped cattle, which might represent the recent introduction of Simmental cattle. To ensure the accuracy of studying Lincang humped cattle, this particular sample was removed in the follow-up analysis.

### Candidate Regions and Genes Under Positive Selection

**FIGURE 3:**
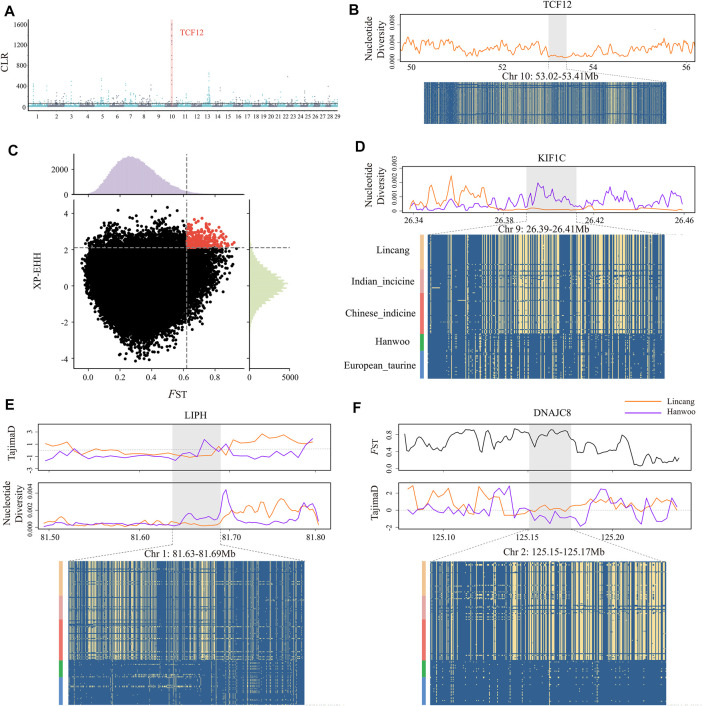
Selective signals in Lincang cattle. **(A)** Manhattan plot of selective sweeps by the CLR method. The horizontal lines indicate the significance threshold (where CLR >61) used for extracting outliers. **(B)** Nucleotide diversity plots and haplotype pattern heatmaps of the *TCF12* gene region. **(C)** Distribution of the *F*_ST_ (x axis) and XP-EHH (y axis) between Lincang and Hanwoo cattle. The dashed vertical and horizontal lines indicate the significance threshold (where *F*_ST_ > 0.62 and XP-EHH > 2.1) used for extracting outliers. **(D)** Nucleotide diversity and degree of haplotype sharing across populations at the *KIF1C* gene region. **(E)** TajimaD, nucleotide diversity, and degree of haplotype sharing across populations at the *LIPH* gene region. **(F)** *F*_ST_, TajimaD, and degree of haplotype sharing across populations at the *DNAJC8* gene region. The major allele at each SNP position is colored in yellow.

The composite likelihood method (CLR) was applied to detect the selection signals in Lincang humped cattle (Figure 3A). The top 1% signal window was selected as candidate regions, while 618 genes were annotated with selection characteristics (Supplementary Table S3). KEGG pathway and gene ontology (GO) analyses were used to perform functional enrichment analysis. However, no significant enrichment pathway was found. Surprisingly, the *TCF12* gene was annotated in the top 10 signal windows of CLR. The primary biological process of *TCF12* is to orchestrate the activity of myogenic factors through myogenic differentiation (Parker, Perry, Fauteux, Berkes, & Rudnicki, 2006). Throughout the entire *TCF12* region, Lincang humped cattle showed low nucleotide diversity and constant haplotype diversity patterns (Figure 3B).

The fixation index (*F*_ST_) test was performed on various groups (I) Lincang humped cattle and Indian indicine; (II) Lincang humped cattle and Chinese indicine; (III) Lincang humped cattle and Hanwoo cattle; and (IV) Lincang humped cattle and European taurine, averaging 0.047, 0.048, 0.32, and 0.34, respectively. Moreover, the *F*_ST_ and XP-EHH methods were performed on Lincang and Hanwoo cattle to detect the significant positive selection signatures, owing to their vast genetic differences between both breeds pertaining to the ecological adaption (Figure 3C). The outlier regions were screened in the top 1% of the empirical distribution of *F*_ST_ and XP-EHH statistics (Supplementary Table S4) and annotated (316 genes). The pathway “negative regulation of protein kinase activity” (FDR = 0.03) was significantly enriched in the KEGG pathway, which might play an important role in the adaptation of Lincang humped cattle to stressful environments.

Moreover, it is noticeable that the regions scanned by *F*_ST_ on BTA1(81.60–81.65 MB), BTA5 (47.58–47.65 MB), BTA7 (43.16–43.27, 43.40–43.45 MB), BTA16 (50.56–50.61 MB), and BTA19 (26.38–26.45 MB) showed a strong positive selection signal, while using XP-EHH, BTA2 (125.14–125.19 MB), BTA7 (44.54–44.63 MB), BTA11 (55.84–55.89 MB), and BTA12 (24.12–24.19, 76.56–76.65 MB) regions showed strong positive selection signals (Table 1). Overall, genes related to the body size (*SENP2*, *KIF1C*, and *PFN1*) (Figure 3D and Supplementary Figure 2A), immunity (*LIPH*, *IRAK3*, *GZMM*, and *ELANE*) (Figure 3E and Supplementary Figure 2B), and heat resistance (*MED16*, *DNAJC8*, and *HSPA4*) (Figure 3F and Supplementary Figure 2C,D) were identified in the 11 candidate genomic regions. Most target genes exhibited lower nucleotide diversity, higher *F*_ST_, and differential Tajima’s D values than Hanwoo genomic regions, indicating strong selective sweeps. Furthermore, a missense mutation (rs521365524) was found in the *IRAK3* gene, an immune-related gene. This mutation presented a predominant divergence between Lincang humped cattle (allele G frequency = 0.9) and Hanwoo cattle (allele T frequency = 1). Another missense mutation (rs210913195) was detected in the heat-related gene *HSPA4*, which showed a widespread pattern in Lincang humped cattle (frequency 0.98) and the opposite pattern in Hanwoo cattle (frequency 0.2).

**TABLE 1:** Genomic regions and associated genes putatively under selection identified using *F*_ST_, XP-EHH, and CLR statistics.

Test	BTA	Start (pb)	End (pb)	Max *F*_ST_	Max XP-ehh	Max-CLR	Genes
Top *F*_ST_	1	81600001	81650000	0.875749	——	——	*SENP2*, *LIPH*
	5	47580001	47650000	0.879839	——	——	*TRNAK-CUU*, *IRAK3*, and *TMBIM4*
	7	43160001	43270000	0.920406	——	——	*MADCAM1*, *TPGS1*,*CDC34*, *GZMM*, *BSG*, *HCN2*, *TRNAE-UUC*, *FGF22*, *POLRMT*, and *RNF126*
	7	43400001	43450000	0.908626	——	——	*PRTN3*, *ELANE*, *R3HDM4*, *CFD*, and *MED16*
	16	50560001	50610000	0.900547	——	——	*SKI*, *PRKCZ*, and *FAAP20*
	19	26380001	26450000	0.890845	——	——	*INCA1, KIF1C*, *CAMTA2*, *SPAG7*, *PFN1*, and *ENO3*
Top XP-EHH	2	125140001	1.25E+08	——	3.26	——	*ATP5IF1* and *DNAJC8*
	7	44540001	44630000	——	3.74	——	*ZCCHC10*, *AFF4*, and *HSPA4*
	11	58840001	58890000	——	3.36	——	*LRRTM4*
	12	24120001	24190000	——	3.66	——	*TRPC4*
	12	76560001	76650000	——	3.57	——	*CLYBL*
*F*_ST_, XP-EHH, and CLR	1	44080001	44250000	0.796722	2.8	443.703861	*CMSS1* and *FILIP1L*
	2	61500001	61570000	0.710715	2.94	82.469142	*LCT* and *MCM6*
	5	47500001	47570000	0.736184	2.64	92.679749	*HELB* and *IRAK3*
	7	90280001	90430000	0.776616	2.68	220.938087	*ADGRV1* and *MIR2464*
	11	73880001	73930000	0.642026	2.17	73.228089	*DTNB*
	13	61160001	61390000	0.781211	2.54	185.266809	*COX4I2*, *ID1*, *BCL2L1*, *TPX2*, *MYLK2*, and *FOXS1*
	19	39780001	39850000	0.674589	2.53	79.785159	*FBXL20*, *TRNAW-CCA*, and *MED1*
	19	44040001	44090000	0.764124	2.22	71.273024	*SLC4A1*

**FIGURE 4:**
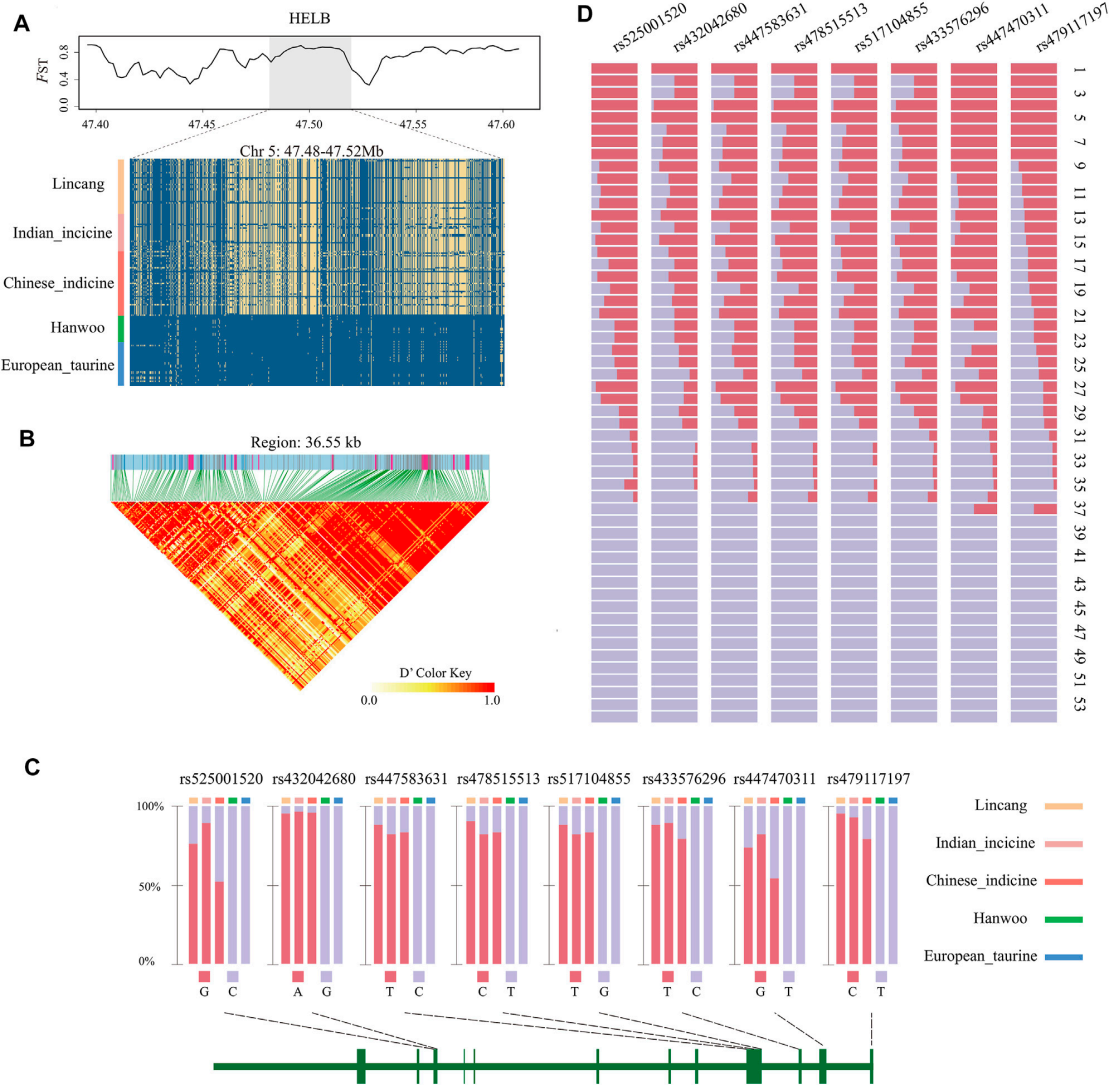
Characteristics of the *HELB* gene. **(A)** *F*_ST_, TajimaD, and degree of haplotype sharing across populations. The major allele at each SNP position is colored in yellow. **(B)** Allele frequency of the eight missense SNPs in each group and the schematic structure of genes. The vertical bars in the structure diagram represent the exon region. **(C)** LD plot of SNPs. LD values (D′) between two loci are detailed in boxes (D′ = 0–1). D′ = 1 indicates perfect disequilibrium. **(D)** Allele frequency of the eight missense SNPs across the 54 populations in the BGVD. Population names associated with serial numbers are as follows. 1 Tharparkar; 2 Sahiwal; 3 Hariana; 4 Brahman; 5 Nelore; 6 Gir; 7 Dabieshan; 8 Wandong; 9 Jinjiang; 10 Lingnan; 11 Shorthorn Zebu; 12 Kenana; 13 Srilanka; 14 Bashan; 15 Dianzhong; 16 Wenshan; 17 Guangfeng; 18 Jian; 19 Weining; 20 Ogaden; 21 Leiqiong; 22 Luxi; 23 Nganda; 24 Boran; 25 JiaxianRed; 26 Kazakh; 27 Wannan; 28 Zaobei; 29 Nanyang; 30 Rashoki; 31 Tibetan; 32 Ankole; 33 NDama; 34 Chaidamu; 35 Mongolian; 36 BohaiBlack; 37 Yanbian; 38 Nsongora; 39 Mishima; 40 Kuchinoshima; 41 Hanwoo; 42 Jerse; 43 Simmental; 44 Gelbvieh; 45 Piedmontese; 46 Limousin; 47 Salers; 48 Charolais; 49 MaineAnjou; 50 Devon; 51 Holstein; 52 Hereford; 53 RedAngus; and 54 Angus.

It is worth noting that eight overlapped genomic regions and 19 genes were detected among the three mentioned selection methods (Table 1), indicating that these were strongly selected in Lincang humped cattle. Among them, *FILIP1L*, *HELB*, *BCL2L1*, and *TPX2* genes were all associated with heat stress. Candidate genes showed a stable haplotype diversity pattern in Lincang humped cattle or discrepant Tajima’s D and nucleotide diversity (Figure 4A and Supplementary Figure S2E,F).

### Eight Missense Mutations in *HELB* to Indicine Cattle

The low diversity of the *HELB* gene haplotype in Lincang humped cattle (Figure 4A), corresponding to the LD heatmap, showed strong linkage disequilibrium (Figure 4B). Surprisingly, we detected eight missense mutations (rs433576296, rs517104855, rs478515513, rs447583631, rs432042680, rs479117197, rs447470311, and rs525001520) that were located within the *HELB* gene, indicating significant genomic differences between Lincang humped cattle and Hanwoo cattle. The allele frequencies of these missense mutations were estimated in the five major cattle populations. These missense mutations only occur in indicine cattle in our samples (Figure 4C), while one of the missense mutations (rs447470311) has been confirmed to be specific to indicine cattle (Naval-Sánchez et al., 2020).

To check whether these mutations are also specific for indicine cattle in a wider cattle breed population, the frequencies of these eight missense mutations were searched among 432 individuals from 54 cattle breeds around the world (Figure 4D, Supplementary Table S5, S3), adopting BGVD (Bovine Genome Variation Database and Selective Signatures) (Chen et al., 2020) (Figure 4D, Supplementary Table S5, S3). It was observed that 37 breeds harbored rs479117197 and rs447470311 mutations, where 36 out of 37 were either indicine breeds or mixed with indicine. The remaining one was Yanbian cattle from Northeast Asia, which may have individual deviations. At the same time, in rs433576296, rs517104855, rs478515513, rs447583631, rs432042680, and rs525001520, only breeds with indicine ancestry showed the mutations, revealing a higher frequency of these mutations in indicine ancestry alone. It is worth mentioning that the species from Mongolian, Chaidamu, Kazakh from northwest China, and Tibetan depicted low-frequency mutations, probably due to minute indicine introgression (Chen et al., 2018).

## Discussion

Lincang humped cattle is one of the groups of Yunnan high-humped cattle in the southern part of Lincang and Pu’er cities bordering Myanmar. Lincang humped cattle has become a valuable genetic resource in the region because of its good environmental adaptability and particularity to the Wa ethnicity (Gan, 2011). As cattle genetic resources are being exhausted (Y. Zhang, 2011), the genetic characterization of Lincang humped cattle is of great significance to this vital genetic resource.

The genetic diversity of genomes may reflect differences in cattle management or breed history. Compared to taurine cattle with long breeding histories, indicine cattle has a lower degree of selection history and higher genetic diversity, consistent with our results and matching those observed in earlier studies (Mei et al., 2018). The Myanmar border is a habitat of Indian indicine, whereas Lincang and Pu’er areas inhabit Lincang humped cattle admixed with Chinese and Indian indicines. Prior studies mark Yunnan as an admixture zone of *Bos taurus* and *Bos indicus* (R. Li et al., 2019), and the entry of Indian indicine in China is also proposed through the Yunnan border (Jia et al., 2007). Our research results indicated that Lincang humped cattle are composed of Indian–Chinese indicine cross genotypes in the genetic structure. This demonstrated that Indian indicine may have entered East Asia through the Lincang and Pu’er areas, which may be an important route for Indian indicine to migrate from the domestication sites (Utsunomiya et al., 2019).

The smaller body size of Lincang humped cattle is associated with its ecological adaptation to hot and humid conditions (Gardner, Peters, Kearney, Joseph, & Heinsohn, 2011). Interestingly, our study detected genes related to skeletal muscle development (*TCF12*, *SENP2*, *KIF1C*, and *PFN1*). Skeletal muscle development is a complex biological process involving multiple key genes. The protein encoded by *TCF12* acts as a complex to positively regulate itself during muscle development (Fu et al., 2020). Related pathways include extracellular signal-regulated kinase signal transduction and CDO in myogenesis (Parker et al., 2006). The *SENP2* gene plays an essential role in the regulation of the muscle growth inhibitor expression and myogenesis, which encodes SUMO-specific protease 2 and is an important regulator of fatty acid metabolism in skeletal muscle (Koo et al., 2015). *KIF1C* plays a role in maintaining membrane circulation during myogenesis and adult muscle (Ginkel & Wordeman, 2000). *PFN1* is a critical factor in skeletal development and regulates sternal bone development and endochondral bone formation (Miyajima et al., 2012). It should be noted that Lincang humped cattle weigh less than 300 kg and have an average height of 1 m (Y. Zhang, 2011). They were small body size cattle. The change in the body size can be explained as an adaptive response to the climate, which means positively selected genes associated with the body size may contribute Lincang humped cattle in humid and hot conditions.

The superior adaptability of Lincang humped cattle is partly attributed to their resistance to disease and parasites (Turner, 1980). Our study detected genes related to immune response and parasite resistance (*LIPH*, *IRAK3*, *GZMM*, and *ELANE*). A previous study identified the association of *LIPH* with cattle immunity (Zhuang et al., 2021). Similarly, the *LIPH* gene is also found in Dehong cattle (the same Yunnan high-humped cattle as Lincang humped cattle) (R. Li R et al., 2020). *IRAK3* is thought to be a negative regulator of innate immune signaling (Lange, Nelen, Cohen, & Kulathu, 2021). In addition, this gene is also found in selective scans of other indicine cattle (Naval-Sánchez et al., 2020). *GZMM* can affect the killing efficacy against intracellular pathogens (S. Wang, Xia, Shi, & Fan, 2012). *ELANE* mutations may trigger neutrophil precursors’ death and lead to neutropenia (Garg et al., 2020). Furthermore, *ELANE* and *GZMM* have also been demonstrated in African N’Dama cattle, depicting multiple biological functions in parasitic infections (Ben-Jemaa et al., 2020).

For indicine cattle that have lived in tropical and subtropical climatic conditions, several reports have shown that the indicine breed exhibits stronger heat tolerance (Hansen, 2004; J.; Kim et al., 2017). This was also demonstrated by our screening of candidate genes for DNA damage repair and apoptosis associated with heat resistance (*MED16*, *DNAJC8*, *HSPA4*, *FILIP1L, HELB*, *BCL2L1*, and *TPX2*). *MED16* is recruited as the *HSP* gene promoter in response to heat stress (S. Kim & Gross, 2013). Previous studies have established that knockdown of *DNAJC8* decreases antioxidant defenses and increases oxidative damage in honeybees, while *DNAJC8* has been shown to function significantly under heat stress in honeybees (G. Li G et al., 2020). Moreover, *HSPA4* promotes cellular protection against thermal damage and prevents protein denaturation (Niu et al., 2006). Furthermore, several recent genome-wide analyses had detected selective scans for *HSPA4* and highlighted it as a candidate gene for adaptation to hot climates in African indicine cattle (Edea et al., 2018; J.; Kim et al., 2017). *FILIP1L* interacts with *HSF1* to modulate the heat shock response (Hu & Mivechi, 2011). *BCL2L1* acts as an anti-apoptotic gene to control apoptosis inducers (Zinkel, Gross, & Yang, 2006), and it has been suggested that it may be a valuable candidate for heat stress studies in dairy cattle (Khan et al., 2020). *TPX2* functions in the amplification of the DNA damage response (Neumayer, Belzil, Gruss, & Nguyen, 2014). Considering that Lincang humped cattle are well-adapted to hot climates, these genes may play a vital role in the thermal adaptability of Lincang humped cattle.

*HELB* is involved in DNA damage response as a DNA end-excision inhibitor (Tkáč et al., 2016). Multiple mutations in *HELB* have been identified in mouse cell lines with temperature-sensitive DNA replication (Tada et al., 2001). Furthermore, the mutation rs447470311 in *HELB* revealed in tropical cattle may allow better adaptation to the environment (Naval-Sánchez et al., 2020). An important finding in our study was not only the identification of rs447470311 specific to cattle with indicine cattle pedigree but also the identification of seven missense mutations (rs479117197, rs433576296, rs517104855, rs478515513, rs447583631, rs432042680, and rs525001520) which were only found in cattle with indicine pedigree. Meanwhile, the strong linkage disequilibrium of *HELB* implied that the significant association of a few SNPs in the gene with the trait may be sufficient to indicate association with the majority of SNPs in the gene and implied a substantial enrichment of the biological function (Qanbari, 2019). Therefore, these results may support the hypothesis that missense mutations in *HELB* caused alterations in its DNA damage response function, making indicine cattle more adapted to the hot environment. Additional studies may be required in the future to fully understand the effects of *HELB* on adaptation in indicine cattle.

## Conclusion

This study explored the genomic variation in the local cattle population at the China–Myanmar border for the first time *via* whole-genome resequencing data. The genomic diversity of Lincang humped cattle was explored and identified as indicine cattle. It is proposed that the Indian indicine might have migrated to southwestern China through the Lincang and Pu’er areas. In addition, we identified candidate genes associated with environmental adaptations such as the body size, immunity, and heat tolerance. Finally, we identified missense mutations in *HELB* that were specific to indicine cattle and were presumed to be associated with adaptation to hot environments. Overall, these results provided a basis for a proper genetic assessment of Lincang humped cattle and further studies on the relationship between *HELB* and heat tolerance in indicine cattle.

## Data Availability

The datasets presented in this study can be found in online repositories. The names of the repository/repositories and accession number(s) can be found in the article/Supplementary Material.

## Supplementary Material

Supplementary Figure S1 Information for each group. **(A)** Locations for each cattle population. **(B)** The specific and shared SNPs for each group. The numbers in the circle components show specific SNPs for each group or overlapping SNPs among groups.

Supplementary Figure S2
*F*_ST_, TajimaD, nucleotide diversity, or degree of haplotype sharing across populations. The major allele at each SNP position is colored in yellow. **(A)** *MED16* gene region **(B)** *IRAK3* gene region **(C)** *SENP2* gene region **(D)** *FILIP1L* gene region **(E)** *HSPA4* gene region **(F)** *BCL2L1* gene region **(G)** *TPX2* gene region.

Supplementary Figure S3 Allele frequency of the eight missense SNPs across the 432 individuals from 54 cattle breeds around the world by the BGVD.
