# Supplementary figures and images for: Whole-Genome Analyses Reveal Genomic Characteristics and Selection Signatures of Lincang Humped Cattle at the China–Myanmar Border
Source: Front Genet. 2022 Mar 22;13:833503. doi: 10.3389/fgene.2022.833503 (PMC8981028; doi:10.3389/fgene.2022.833503)

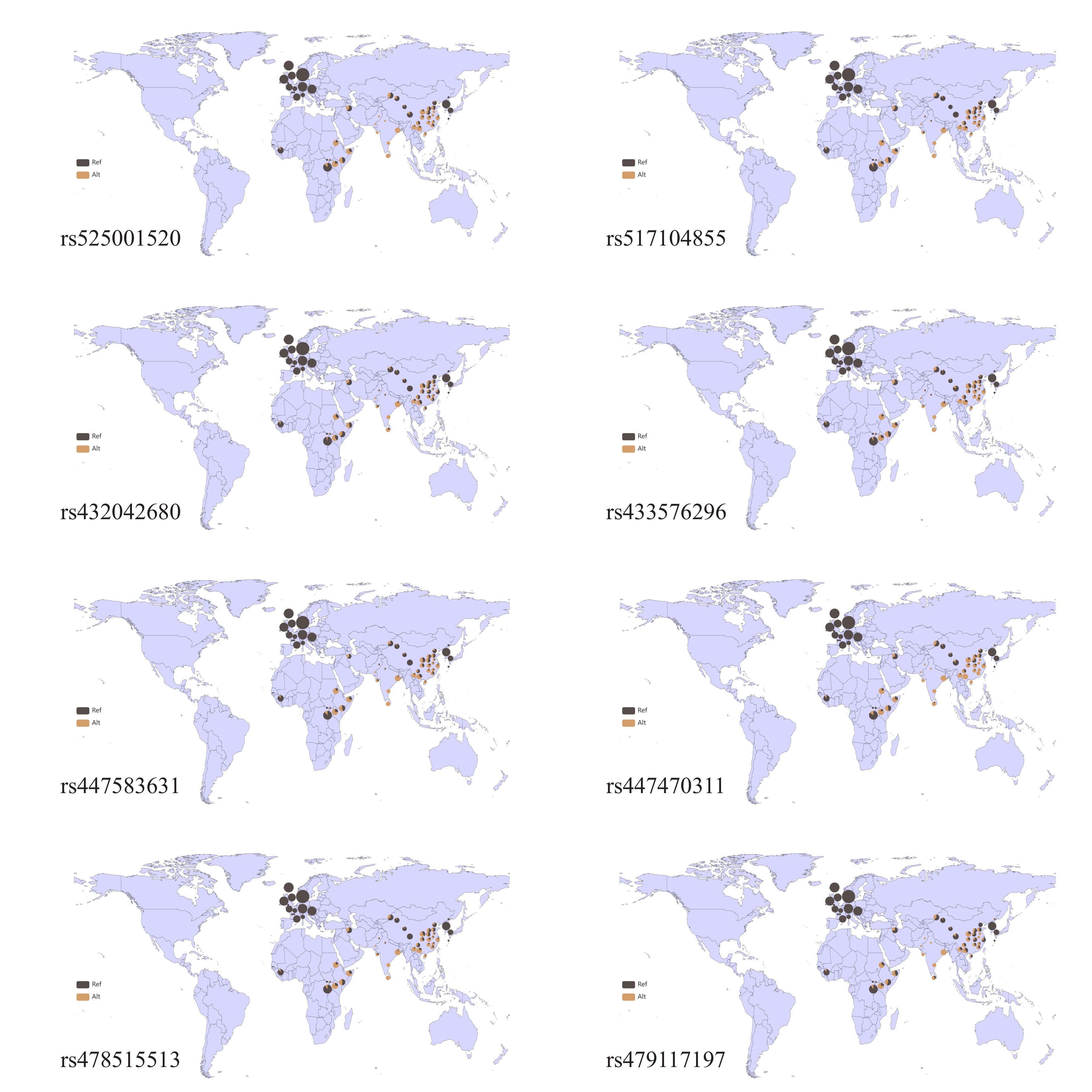

Supplement: Supplementary file 1 [file Image1.TIF]

**A**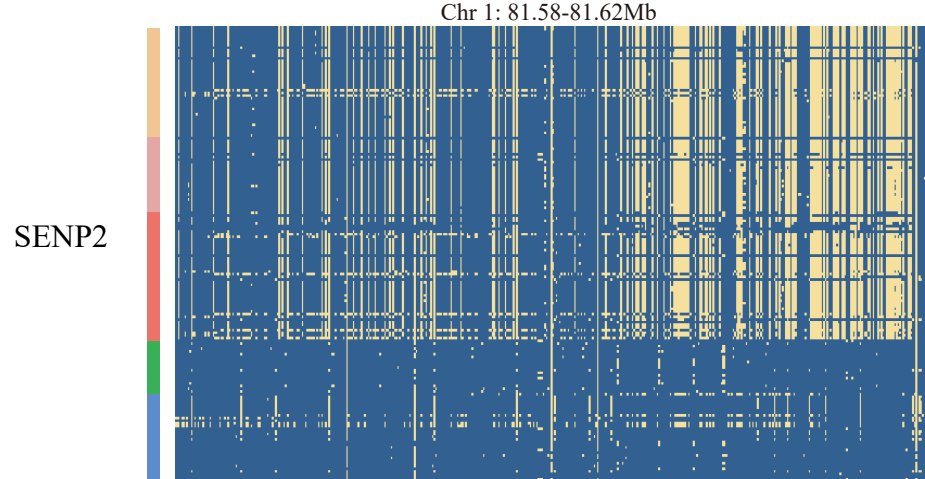**B**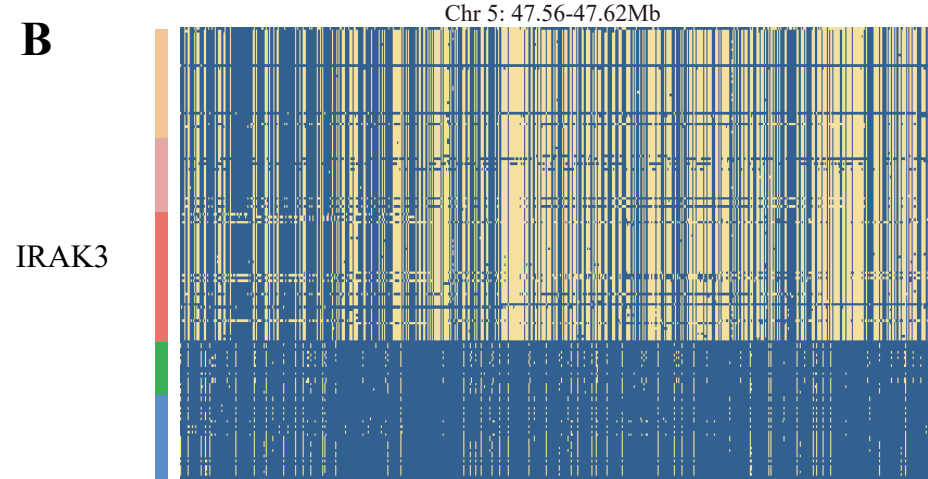**C**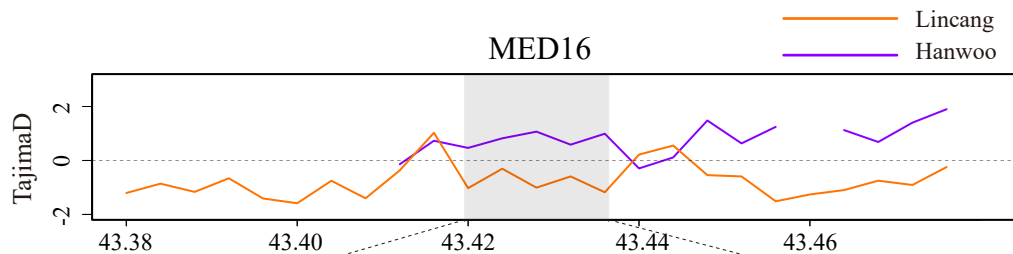**D**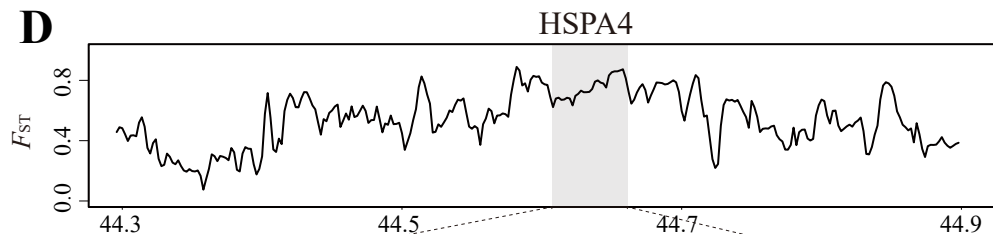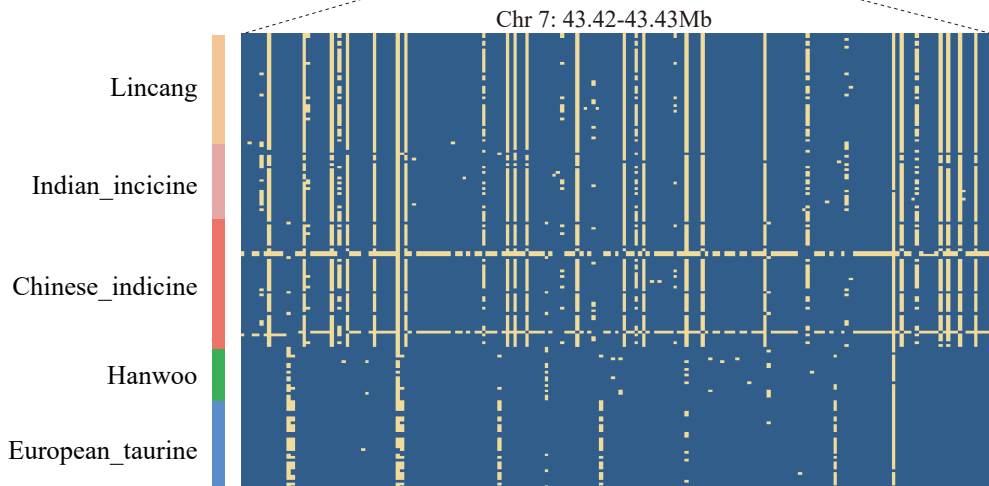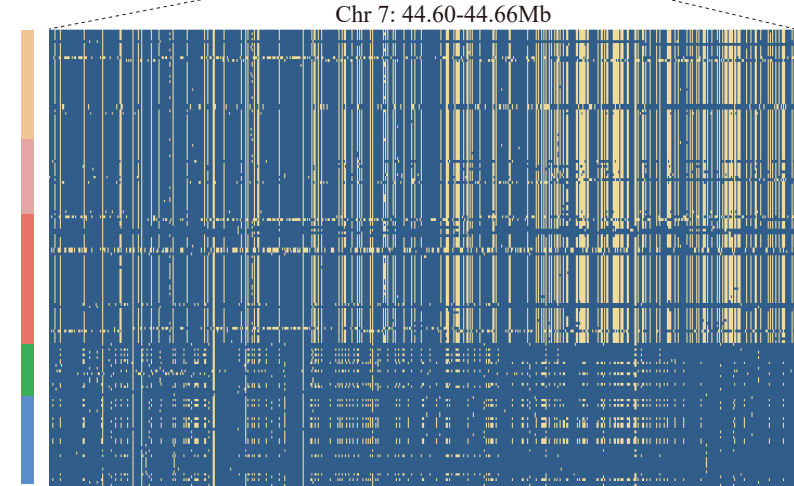**E**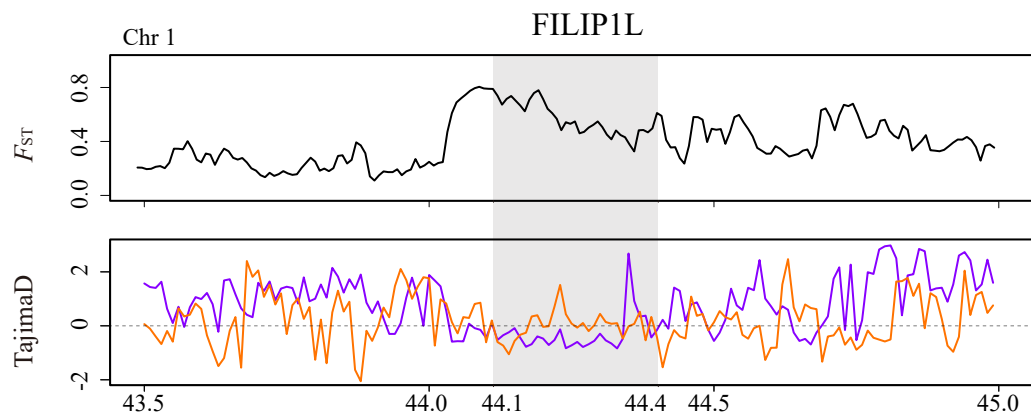**F**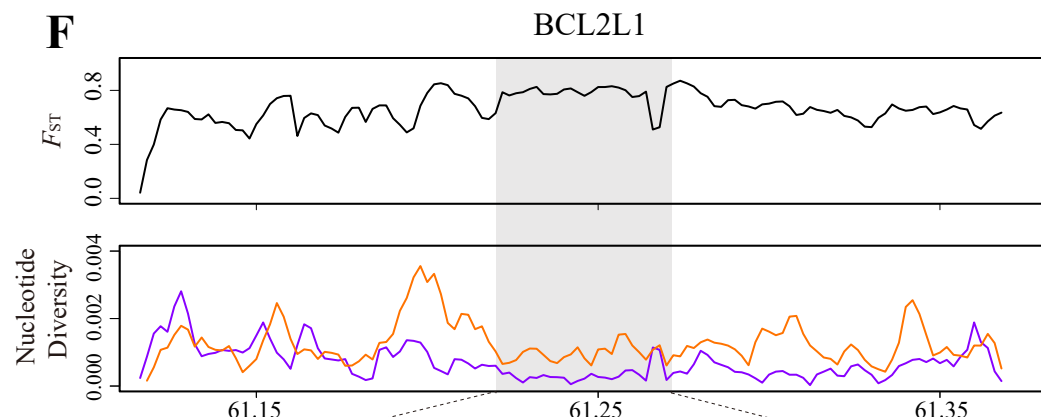**G**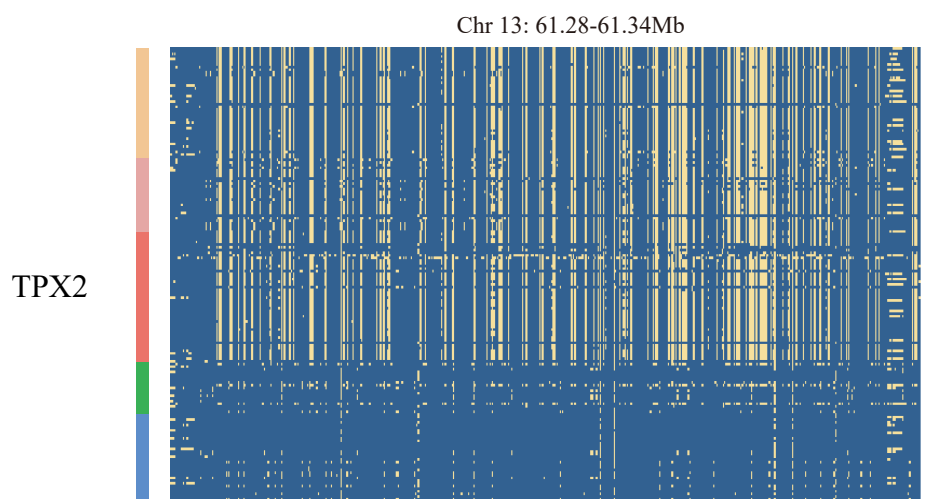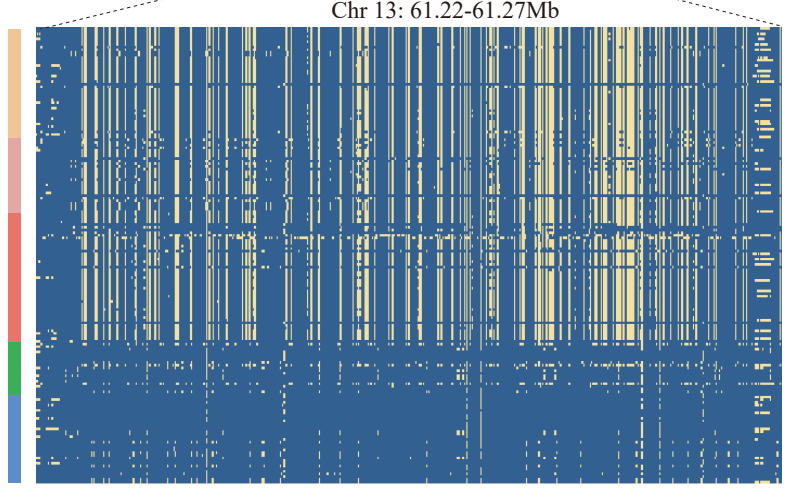

Supplement: Supplementary file 3 [file Image3.PDF]
