# Supplementary material for: Whole-Genome Analyses Reveal Genomic Characteristics and Selection Signatures of Lincang Humped Cattle at the China–Myanmar Border
Source: Front Genet. 2022 Mar 22;13:833503. doi: 10.3389/fgene.2022.833503 (PMC8981028; doi:10.3389/fgene.2022.833503)
Supplement: Supplementary file 2 [file Image2.PDF]

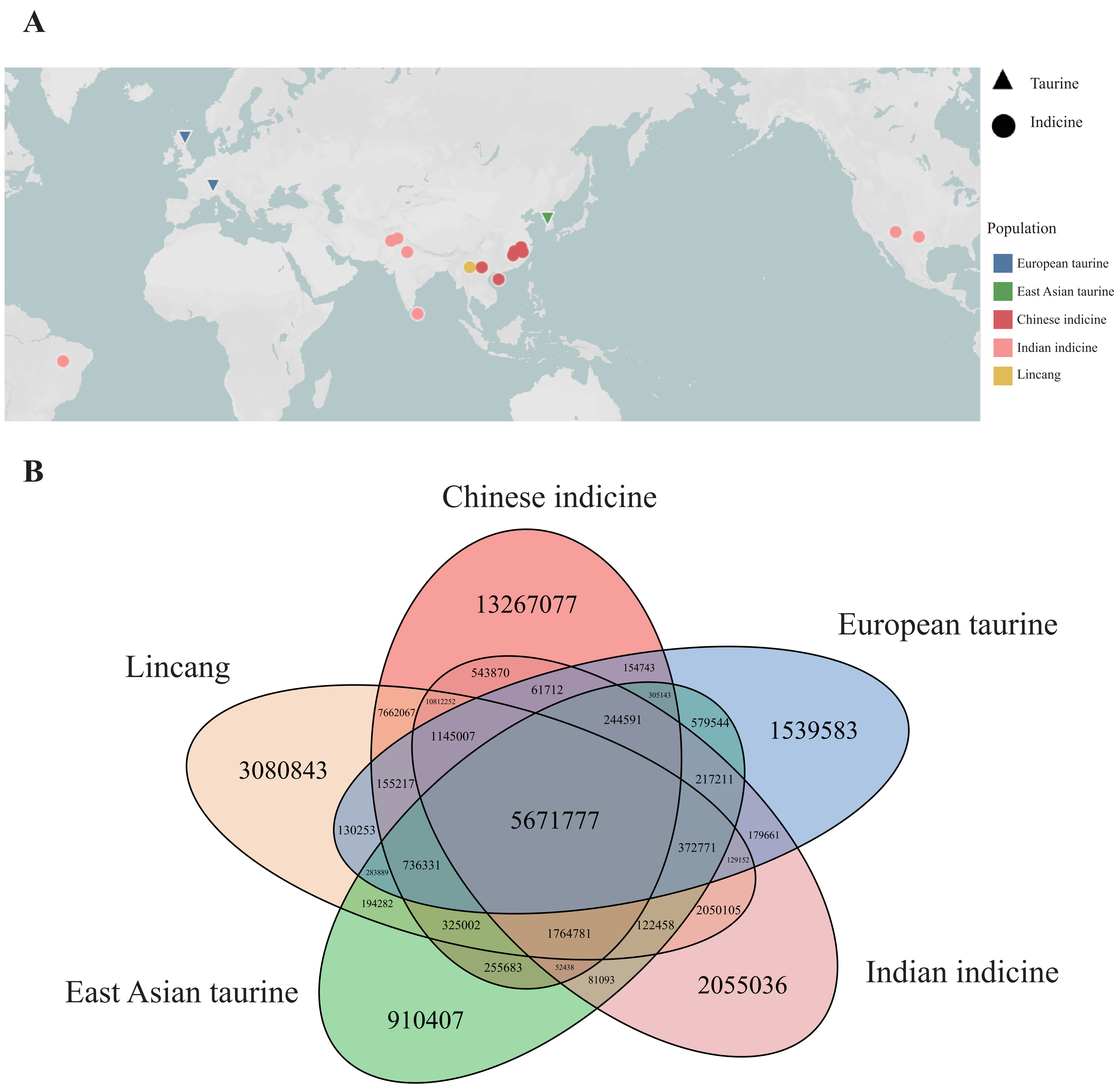
A
Taurine
Indicine
Population
European taurine
East Asian taurine
Chinese indicine
Indian indicine
Lincang
B
Chinese indicine
European taurine
Lincang
East Asian taurine
Indian indicine
13267077
1539583
3080843
910407
2055036
5671777
543870
154743
61712
10812252
305143
7662067
244591
579544
1145007
155217
217211
130253
179661
372771
129152
736331
283889
194282
2050105
325002
122458
1764781
255683
52438
81093
